# Prescription Patterns in Rheumatoid Arthritis Patients at a Tertiary Care Hospital: A Prospective Cross-Sectional Study

**DOI:** 10.7759/cureus.110431

**Published:** 2026-06-08

**Authors:** Kaushalendra Mani, Sujata Jadhav, Vandana Thorat, Prathmesh Gargate, Sharad Tat

**Affiliations:** 1 Department of Pharmacology, Krishna Institute of Medical Sciences, Krishna Vishwa Vidyapeeth (Deemed to be University), Karad, IND

**Keywords:** deflazacort, diclofenac, dmards, methotrexate, rheumatoid arthritis

## Abstract

Introduction: Rheumatoid arthritis (RA) is a chronic, debilitating, systemic inflammatory condition seen worldwide. Prescribing medications is one of the most common interventions in RA treatment. Therefore, we aimed to study the prescription pattern for RA patients at our tertiary care center.

Methods: This was a prospective, cross-sectional, descriptive study of 95 RA patients. Data on demographic profile and drug prescriptions were collected from the prescriptions given by consultants and then analyzed.

Results: Most patients were aged 41-60 years (66.3%), and females predominated (61.1%). Common comorbidities included hypertension (21.1%), diabetes (16.8%), and anemia (12.6%). Disease duration was mainly three to five years (55.8%). Nearly half had an education below graduation (49.5%); common occupations were housewives (37.9%) and professionals (32.6%). Disease‑modifying anti‑rheumatic drugs (DMARDs) were the most prescribed class (90.5%), followed by nonsteroidal anti‑inflammatory drugs (NSAIDs; 64.2%) and corticosteroids (51.6%); biologics were limited (8.4%). Three‑drug prescriptions were most common (35.8%). Methotrexate was the predominant DMARD (75.8%), while diclofenac (20.0%) and deflazacort (18.9%) were the most frequently used NSAID and corticosteroid, respectively.

Conclusion: RA predominantly affects middle‑aged females with moderate disease duration and a notable comorbidity burden. Treatment patterns show strong reliance on conventional DMARDs, particularly methotrexate, with adjunct use of NSAIDs and corticosteroids, while biologics remain underused.

## Introduction

Rheumatoid arthritis (RA) is a chronic, disabling systemic inflammatory disorder of unknown cause. It usually presents symmetrical, progressive, and erosive polyarthritis affecting both small and large synovial joints, along with various extraarticular features [1]. The small joints of the hands, wrists, and ankles are commonly involved, typically as symmetric polyarthritis [2]. Clinically, patients have joint swelling, pain, and morning stiffness lasting more than one hour [3]. Without appropriate treatment, ongoing inflammation leads to progressive joint damage, including cartilage destruction and bone erosion [4,5]. Over time, these structural changes result in permanent joint deformities and significant functional disability, impairing the patient’s quality of life [6,7]. 

RA is a global health concern affecting all ethnicities, age groups, and both sexes. In India, its prevalence is about 0.75% among adults. Incidence rises between 25 and 55 years of age, stabilizes up to around 75 years, and then declines. Women are affected two to 3.5 times more often than men, a difference largely attributed to hormonal influences that diminish with age, especially by the sixth decade [8]. 

Prescribing medication is one of the most frequent therapeutic interventions in clinical practice [9]. Over recent decades, major advances have been made in RA management, driven by a better understanding of disease mechanisms and the introduction of targeted therapies, including monoclonal antibodies and small molecule inhibitors [1]. In 2015, the American College of Rheumatology (ACR) updated its guidelines for RA management, recommending conventional synthetic disease‑modifying anti‑rheumatic drugs (csDMARDs), biologic DMARDs (bDMARDs), targeted synthetic DMARDs (tsDMARDs), and glucocorticoids for both early and established disease [10]. Despite newer agents, methotrexate remains the cornerstone of therapy [1]. 

Currently, csDMARDs such as methotrexate, sulfasalazine, hydroxychloroquine, and leflunomide are the primary agents used worldwide, especially in developing countries like India, where economic constraints limit access to biologic therapies and comprehensive health insurance. Available bDMARDs in India include anti‑TNF agents (etanercept, infliximab, adalimumab, and golimumab), anti‑B cell therapy (rituximab), anti‑IL‑6 therapy (tocilizumab), and co‑stimulation modulators (abatacept) [11]. 

Drug utilization studies are essential for evaluating prescribing patterns, identifying irrational drug use, and providing feedback to clinicians, thereby promoting rational prescribing and improving patient care [12]. With this in mind, we conducted the present study to examine the prescription pattern for RA patients at our tertiary care center. 

## Materials and methods

Study design and population 

The present study was conducted in the Orthopaedic and Medicine outpatient department in Krishna Charitable Hospital & Medical Research Center, Krishna Institute of Medical Sciences, Krishna Vishwa Vidyapeeth (Deemed to be University), Karad, India, after taking approval from the Institutional Ethics Committee (Protocol No. 351/2023-2024; Ref No. KVV/IEC/05/2024; dated 18 April 2024).

The study was conducted between May 2024 and December 2025.

Study type

This was a prospective cross-sectional descriptive study involving 95 patients with RA diagnosed using two blood tests, viz., rheumatoid factor (RF) and anti-CCP. The consecutive sampling method was followed.

The sample size was calculated using the following formula for estimating a proportion.

$n=\frac{4pq}{l^2}$

where p is the expected prescription frequency of DMARDs combination (methotrexate + hydroxychloroquine) (64%), q = 100 - p (36%), and l is the allowable error (10%). Substituting these values, the minimum sample size obtained was 92, and hence the final sample size was rounded up to 95.

Inclusion and exclusion criteria 

Patients aged more than 18 years, irrespective of gender, who had been diagnosed with RA and had received drug therapy for the same for at least six months were included in the study. Patients were excluded if they were pregnant women, had end-stage organ failure, cognitive impairment, or any other type of arthritis apart from RA. 

Data collection 

Institutional Ethics Committee approval was obtained prior to the initiation of the study. Written informed consent was taken before enrolling the patient in the study. The study protocol and informed consent form were also approved by the ethics committee.

Patients diagnosed with RA and fulfilling the inclusion and exclusion criteria were included in the study.

Demographic data (age, gender, education, occupation), prescription data (pharmacological class of drug, number of drugs), and other patient details, like duration of disease and comorbidities, were recorded in a patient information sheet and entered into a specially designed clinical report form. Each prescription was then studied and analyzed. Patient was allowed to continue his/her routine treatment. There will be no intervention in the treatment.

Statistical analysis 

Data was entered in Microsoft Excel 2021 (Microsoft Corp., Redmond, WA, USA), and analysis was done using IBM SPSS Statistics software, version 21 (IBM Corp., Armonk, NY, USA). Categorical variables were represented in the form of percentages and frequencies. 

## Results

**Figure 1:**
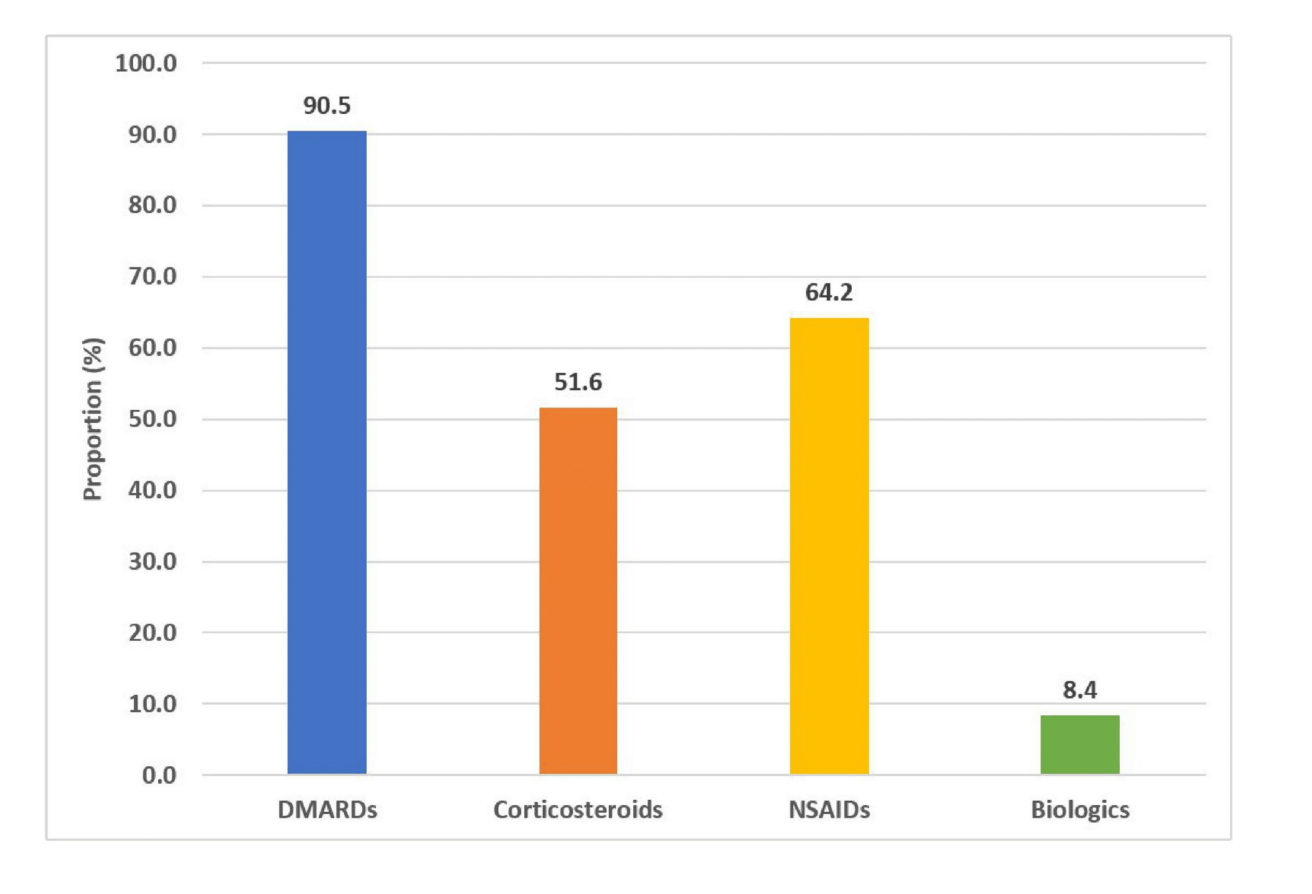
Drug class prescription pattern DMARDs: disease‑modifying anti‑rheumatic drugs; NSAIDs: nonsteroidal anti‑inflammatory drugs

Figure 1 shows DMARDs were the most commonly prescribed drug class (90.5%, 86 patients), followed by NSAIDs (64.2%, 61 patients) and corticosteroids (51.6%, 49 patients). Biologics were prescribed to only eight patients (8.4%).

**Figure 2:**
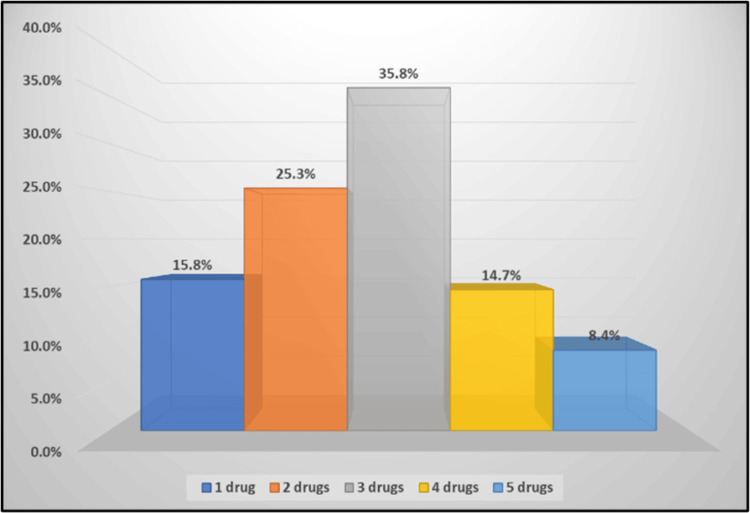
Number of drugs per prescription

Figure 2 shows that three drugs per prescription were most common (35.8%, 34 patients), followed by two drugs (25.3%, 24 patients), and four drugs per prescription were seen in 14.7% (14 patients) of cases. Polypharmacy (five or more drugs) was seen in 8.4% (8) of cases. Only (15.8%, 15 patients) received monotherapy. 

Table 1 shows the socio‑demographic characteristics. Most patients (66.3%, 63 patients) were in the age group 41-60 years, with female predominance (61.1%, 58 patients). The top three comorbidities were hypertension (21.1%, 20 patients), diabetes (16.8%, 16 patients), and anemia (12.6%, 12 patients). Disease duration was three to five years in the majority (55.8%, 53 patients), followed by >5 years (27.4%, 26 patients) and ≤2 years (16.8%, 16 patients). Most patients had less than a high school education (49.5%, 47 patients). The main occupations were housewife (37.9%, 36 patients) and professional (32.6%, 31 patients). 

**Table 1:** Socio‑demographic characteristics of rheumatoid arthritis patients (N=95)

Variable	n (%)
Age (years)	
≤40	11 (11.6)
41–60	63 (66.3)
>60	21 (22.1)
Gender	
Male	37 (38.9)
Female	58 (61.1)
Comorbidity	
Diabetes	16 (16.8)
Hypertension	20 (21.1)
Thyroid disorders	6 (6.3)
Anemia	12 (12.6)
Dyslipidemia	7 (7.4)
Chronic kidney disease	3 (3.2)
Disease duration	
≤2 years	16 (16.8)
3–5 years	53 (55.8)
>5 years	26 (27.4)
Occupation	
Business	16 (16.8)
Daily wages	5 (5.3)
Housewife	36 (37.9)
Professional	31 (32.6)
Retired	7 (7.4)
Education	
Below graduate	47 (49.5)
Graduate	37 (38.9)
Post‑graduate	11 (11.6)

Table 2 summarizes the prescription pattern. Among DMARDs, methotrexate was the most common (75.8%, 72 patients), followed by hydroxychloroquine (50.5%, 48 patients), sulfasalazine (8.4%, eight patients), leflunomide (6.3%, six patients), and azathioprine (2.1%, two patients). 

**Table 2:** Prescription pattern of drug classes (N=95) DMARDs: disease‑modifying anti‑rheumatic drugs; NSAIDs: nonsteroidal anti‑inflammatory drugs

Drug class / Drug	n (%)
DMARDs	
Methotrexate	72 (75.8)
Hydroxychloroquine	48 (50.5)
Sulfasalazine	8 (8.4)
Leflunomide	6 (6.3)
Azathioprine	2 (2.1)
Corticosteroids	
Prednisolone	17 (17.9)
Methylprednisolone	10 (10.5)
Deflazacort	18 (18.9)
Hydrocortisone	4 (4.2)
NSAIDs	
Diclofenac	19 (20.0)
Aceclofenac	5 (5.3)
Etoricoxib	14 (14.7)
Indomethacin	18 (18.9)
Ibuprofen	7 (7.4)
Naproxen	5 (5.3)
Biologics	
Adalimumab	3 (3.2)
Etanercept	3 (3.2)
Rituximab	2 (2.1)

Among corticosteroids, deflazacort (18.9%, 18 patients) was most prescribed, then prednisolone (17.9%, 17 patients), methylprednisolone (10.5%, 10 patients), and hydrocortisone (4.2%, four patients). 

Among NSAIDs, diclofenac (20.0%, 19 patients) was the most common, followed by indomethacin (18.9%, 18 patients), etoricoxib (14.7%, 14 patients), ibuprofen (7.4%, seven patients), aceclofenac (5.3%, five patients), and naproxen (5.3%, five patients). 

Among biologics, adalimumab and etanercept were each prescribed to (3.2%, three patients), and rituximab to (2.1%, two patients). 

## Discussion

Drug utilization studies help evaluate current prescribing trends, identify potential problems, and provide feedback to prescribers, thereby promoting rational prescribing [8]. This study at a tertiary care hospital in Karad, Maharashtra, India, included 95 RA patients over eight months and systematically analyzed their prescription patterns. 

Socio‑demographic findings 

Most patients were aged 41-60 years (66.3%), consistent with reports by Mittal et al. [1] and Patro et al. [13], who reported that RA predominantly affects middle‑aged individuals. Female predominance (61.1%) matches other Indian studies [13, 14] and is explained by estrogen‑mediated immune modulation and X‑chromosome factors [15, 16]. 

Hypertension (21.1%) was the most common comorbidity, followed by diabetes (16.8%) and anemia (12.6%), similar to previous Indian reports [17, 18]. Disease duration of three years in most patients (55.8%) indicates established rather than early RA, comparable to Aslam et al. [19] and Khera [20]. Housewives (37.9%) and professionals (32.6%) were the main occupational groups, similar to some studies [19, 21] but not all [13]. Nearly half of the patients had an education below graduation, supporting findings that RA burden is higher among lower socioeconomic groups in India [14, 22]. 

Drug prescription patterns 

We have analysed the antirheumatoid drugs given (monotherapy and combination) to the patients who are diagnosed with RA.

DMARDs were prescribed most frequently (90.5%), followed by NSAIDs (64.2%) and corticosteroids (51.6%), in line with previous reports [8,23]. Low biologic use (8.4%) matches the findings of Jasani et al. and Shini et al. [24,25] and reflects cost constraints. Three‑drug regimens were most common (35.8%), with an average of 2.75 drugs per prescription, lower than reported by Laxmi Prabha et al. (4.97) [23] and Deshmukh et al. (4.98) [26]. 

Methotrexate was prescribed in 75.8% of patients, reaffirming its cornerstone role, consistent with Dutta et al. (76%) [8]. Its affordability makes it ideal for the Indian population. Hydroxychloroquine (50.5%) was often combined with methotrexate [23]. Sulfasalazine (8.4%) and leflunomide (6.3%) were used less [27], and azathioprine was rare (2.1%) [28]. 

Among corticosteroids, deflazacort was most prescribed (18.9%), followed by prednisolone (17.9%), similar to Dutta et al. [8]. For NSAIDs, diclofenac (20.0%) was most common due to efficacy, availability, and low cost [16]; indomethacin (18.9%) and etoricoxib (14.7%) were also frequently used [8, 23]. 

Biologic use was very low (8.4%), with adalimumab and etanercept each used in 3.2% [28]. This contrasts with the United States, where 60%-80% of RA patients receive biologics, highlighting regional disparities in access and resources. 

Limitations 

This single‑center study may not be generalizable to the broader community. The sample size is small, and the findings cannot be generalized to a larger population. The cross‑sectional design cannot assess temporal relationships, medication adherence, or cost variations. This study also lacks disease activity assessment, an absence of treatment outcome, no evaluation of prescribing appropriateness or guideline adherence, and limited information on drug combinations, dosages, and duration of therapy. Longitudinal studies are needed to evaluate changes over time and the direct effects of interventions. 

## Conclusions

RA predominantly affects middle‑aged females, with the majority presenting moderate disease duration and a significant burden of comorbidities such as diabetes and hypertension. The prescribing patterns observed in this study demonstrate a strong reliance on conventional DMARDs, particularly methotrexate, which remains the cornerstone of therapy. Adjunctive use of NSAIDs and corticosteroids is common, reflecting their role in symptom control and bridging therapy. However, biologics remain markedly underused. Furthermore, individualized therapy based on disease activity, comorbidity profile, and patient preference should be prioritized to optimize outcomes.
